# Publication Times and Impact Factors (IFs) in Dentistry Journals

**DOI:** 10.7759/cureus.32680

**Published:** 2022-12-19

**Authors:** Tomasz Skrzypczak, Anna Skrzypczak, Małgorzata Skrzypczak

**Affiliations:** 1 Faculty of Medicine, Wroclaw Medical University, Wroclaw, POL; 2 Faculty of Dentistry, Wroclaw Medical University, Wroclaw, POL; 3 General Dentistry, Private Dental Practice, Krobia, POL

**Keywords:** journals, clinical dentistry, publication times, bibliometric analyis, peer reviews

## Abstract

Introduction: The speed of manuscript publication in reputable journals plays a crucial role in spreading scientific novelties and may influence the number of received citations. In the present study, the authors investigated the publication speed of dentistry journals. This is crucial for both authors, who desire rapid dissemination of their findings, and patients in need, who seek new therapies.

Materials and methods: This was a cross-sectional bibliometric analysis of published dentistry journals. A list of dentistry journals featured in the 2021 Journal Citation Report was downloaded. A total of five random original articles were extracted from each of these journals. These articles were published between January and December 2020. Median and interquartile range (IQR) times from submission to acceptance, publication in print, online publication, time from acceptance to in print and online publication were calculated. The correlation between publication times and journal impact factor (IF) was examined.

Results: A total of 89 journals were included. Individual time from submission to acceptance (peer review time) ranged from 6 to 279 days, the combined median peer review time was 115 (80-159) days. The overall median time from acceptance to online or print publication was 17 (12-38) and 153 (92-249) days, respectively. Journals with available data concerning publication times tended to have higher IF than others. Only journals that did not have available time from acceptance to online publication had higher IF. There were negative correlations between times from submission (r = -0.442, *p* = 0.007), acceptance (r = -0.616, p* *< 0.001) to in-print publication, and IF. There were no correlations between IF and time from submission to acceptance, acceptance to online publication, and submission to online publication.

Conclusions: Publication times availability was revealed to be an indicator of higher impacted journals, which is a potential new exponent of journal quality. Higher IF values were associated with shorter times from submission to acceptance and in-print publication, which is consistent with current editorial policies.

## Introduction

The process of publishing research changed tremendously over the past years. Publications in highly regarded journals became an indicator of research quality and productivity. Dissemination of research findings within the scientific community gives a possibility to contribute to the evolution of particular fields and convert scientific experiments into hands-on practice [1]. This holds true across multiple research disciplines and dentistry is no exception.

The publishing process has quickly evolved over the last few years. Submission of paper-written manuscripts was replaced by an electronic manuscript processing system. Instead of mailing manuscript proposals, instant electronic systems give the authors insight into each stage of the publication process [1-4]. Although this innovation reduced the time of the publishing process, the total time to publication remained long [1-4]. Exceptional work done by both authors and journals is essential for each paper from submission to publication. To enhance this process, authors could deliver better manuscripts and respond quickly to revisions.

Delay in the introduction of crucial innovations has a negative outcome for patients who need new therapies [1, 5-6], journals in terms of impact factor (IF) [1, 7], and recognition of authors’ scientific work [1-2, 7]. The speed of the publication process, topic fit, and journal quality were found to be the most critical factors that influence an author’s choice of journal [1, 8-9]. It seems reasonable that long publication times could influence an author’s decision to publish in a particular journal [1-2]. However, faster turn-around times which would be acceptable for authors, should not come with lower peer-review quality [1, 10]. Bibliometric analysis of journals critically analyzes and helps to understand the different phases of the publican process in a particular field [1-2, 8, 11]. To the knowledge of the authors, time from submission to acceptance in dentistry journals has not been investigated. The aim of this study is to get insight into the time required for manuscripts to be accepted and published in dentistry journals in 2020. In addition, correlations between publication times and IF were analyzed.

## Materials and methods

From the Thomson Reuters website, a list of dentistry journals, indexed in the Journal Citation Report 2021 was downloaded. All journals under the subject category dentistry, oral surgery, and medicine in Science Citation Index Expanded edition were selected. Journals that published only review articles were excluded. From each of these journals, a total of five random original articles published between January and December 2020 were extracted. Authors selected articles at their own discretion, with no specific randomization method. To reduce bias, articles were selected directly from the journal’s articles lists without a prior publication speed check. The selection process relied only on the article’s titles and dates of final publication. Review articles, correspondence, and editorials were excluded. The authors collected information about the dates of submission, final acceptance, and online and in-print publication from the final versions of the articles. In accordance with previous studies, the date of submission denotes the date of when a manuscript was submitted for consideration for publication in a journal, the date of acceptance denotes the date of communication of the final decision to the corresponding author of a manuscript, dates of in-print and online publication refer to the date of publication of the manuscript online or in-print [1-2]. The periods from submission to acceptance, from acceptance to online publication and in-print, and from submission to online publication and in-print were calculated for each journal [1-2]. The median and interquartile ranges (IQRs, 25%-75%) of each parameter were calculated. Data were examined for normality with the Shapiro Wilk test. Continuous variables were compared with the Mann-Whitney U test. Spearman correlations between IF and publication times were calculated. JASP 0.16.3 (the University of Amsterdam, The Netherlands) software was used for the statistical analysis. p < 0.05 was considered statistically significant.

## Results

**Figure 1:**
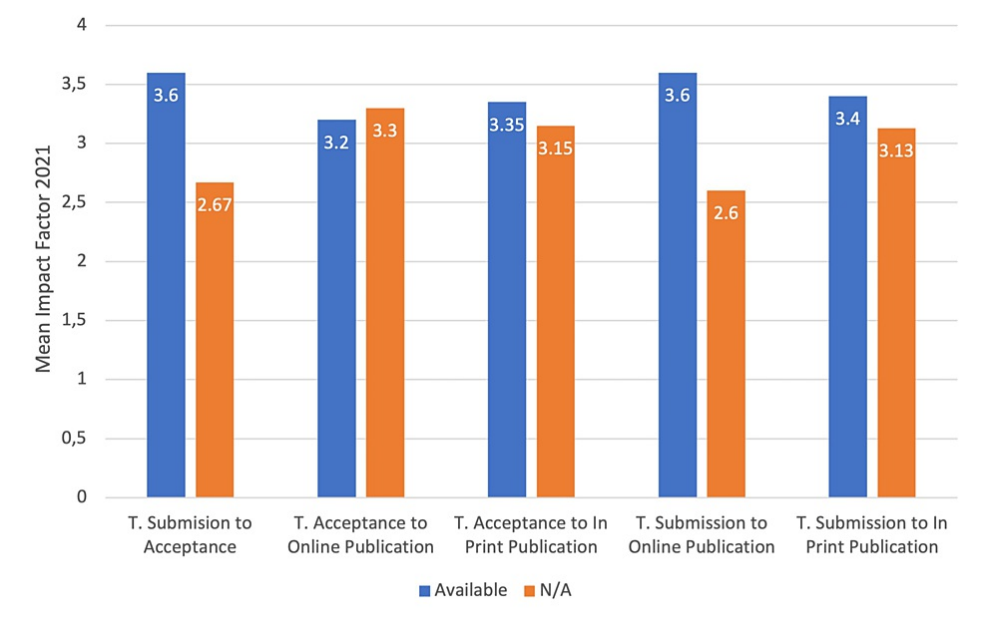
Differences between mean IF and availability of publication times. All differences were statistically significant, p < 0.05; t, time from; N/A, not available IF, impact factor

A total of 89 journals were included. Time from submission to acceptance was unavailable for 34 (38%) journals, time from acceptance to online publication for 31 (35%) journals, time from acceptance to in-print publication for 50 (56%) journals, time from submission to online publication for 34 (38%) journals, and time from submission to in-print publication for 53 (60%) journals. Journals with available data concerning publication times had higher IF than others. However, journals that did not have available time from acceptance to online publication had higher IF. All differences were statistically significant (all p < 0.05, with Mann-Whitney U test) and presented in Figure 1.

Individual time from submission to acceptance (peer review time) ranged from 6 to 279 days, the combined median peer review time was 115 (80-159) days. The overall median time from acceptance to online or print publication was 17 (12-38) and 153 (92-249) days, respectively. Table 1 shows the median publication and peer review time delay for manuscripts in each of the journals.

**Table 1:** Publication and peer review time delay in dentistry journals in 2021. AT Time, median time between submission and acceptance (interquartile range), days; AOP time, median time between acceptance and online publication (interquartile range), days; AIP time, median time between acceptance and in-print publication (interquartile range), days; SOP time, median time between submission and online publication (interquartile range), days; SIPP time, median time between submission and in-print publication (interquartile range), Days; IF, impact factor; N/A, not available; IQR, interquartile range

Journal	AT Time, median (IQR), days	AOP time, median (IQR), days	AIP time, median (IQR), days	SOP time, median (IQR), days	SIPP time, median (IQR), days	IF 2021
Int J Oral Sci	179 (177-223)	52 (30-58)	N/A	209 (200-308)	N/A	24.897
J Dent Res	N/A	N/A	N/A	N/A	N/A	8.924
J Clin Peridontol	47 (46-101)	15 (12-16)	81 (59-83)	63 (58-111)	130 (127-160)	7.478
Oral Onncol	88 (71-122)	13 (9-18)	N/A	106 (78-135)	N/A	5.972
Dent Mater	134 (92-178)	17 (13-18)	61 (61-76)	146 (129-195)	195 (170-239)	5.687
Int Endod J	120 (116-135)	1 (1-2)	77 (63-94)	121 (117-137)	210 (183-212)	5.165
J Evid-Based Dent Pr	146 (122-168)	18 (7-37)	89 (77-103)	164 (149-175)	245 (235-261)	5.100
Clin Oral Implan Res	246 (237-311)	15 (15-63)	113 (92-142)	259 (252-374)	338 (329-453)	5.021
J Dent	101 (96-131)	6 (6-7)	27 (20-39)	107 (103-137)	135 (113-151)	4.991
J Peridontol	N/A	N/A	N/A	N/A	N/A	4.494
J Endodont	N/A	N/A	N/A	N/A	N/A	4.422
J Prosthodont Res	N/A	N/A	N/A	N/A	N/A	4.338
J Adhes Dent	N/A	N/A	N/A	N/A	N/A	4.309
Clin Implant Dent R	102 (94-108)	23 (15-26)	94 (94-98)	121 (121-125)	196 (192-212)	4.259
J Prosthet Dent	N/A	N/A	N/A	210 (210-210)	N/A	4.148
Mol Oral Microbiol	49 (48-62)	8 (8-8)	69 (57-70)	57 (56-70)	119 (105-119)	4.107
Oral Dis	93 (79-118)	11 (7-12)	340 (340-340)	104 (96-130)	433 (419-468)	4.068
J Periodontal Res	73 (23-94)	17 (11-20)	127 (120-140)	93 (40-111)	234 (140-240)	3.946
Caries Res	212 (171-224)	85 (67-117)	137 (130-165)	287 (283-297)	347 (344-349)	3.918
BMC Oral Health	127 (81-175)	10 (9-13)	N/A	170 (93-181)	N/A	3.747
J Dent Sci	156 (156-156)	18 (18-18)	58 (58-58)	24 (17-59)	73 (68-131)	3.719
Int J Oral Impl	N/A	N/A	N/A	N/A	N/A	3.654
Clin Oral Invest	229 (159-310)	10 (9-14)	N/A	243 (168-317)	N/A	3.606
J Oral Rehabil	171 (163-259)	17 (9-18)	63 (63-74)	188 (169-307)	245 (226-349)	3.558
J Oral Pathol Med	62 (50-135)	18 (9-33)	111 (108-189)	95 (78-153)	170 (144-357)	3.539
Dentomaxillofac Rad	23 (13-29)	13 (13-15)	176 (173-184)	36 (28-37)	200 (197-202)	3.525
J Prosthodont	N/A	5 (3-5)	244 (153-260)	N/A	N/A	3.485
J Am Dent Assoc	63 (42-98)	71 (14-100)	150 (137-277)	112 (56-184)	247 (201-352)	3.454
Dent Traumatol	61 (37-188)	26 (25-29)	141 (141-163)	86 (68-204)	202 (190-329)	3.328
Int J Paediatr Dent	78 (76-125)	9 (7-16)	284 (315-385)	100 (88-128)	456 (449-463)	3.264
Prog Orthod	104 (80-107)	30 (29-55)	N/A	134 (125-136)	N/A	3.247
J Cranio Maxill Surg	279 (202-315)	6 (6-7)	168 (168-178)	285 (212-322)	483 (380-509)	3.192
J Appl Oral Sci	82 (74-106)	124 (102-124)	N/A	198 (181-230)	N/A	3.144
Eur J Orthodont	N/A	N/A	N/A	N/A	N/A	3.131
J Esthet Restor Dent	99 (90-122)	15 (13-17)	132 (132-137)	116 (101-139)	236 (234-258)	3.040
Implant Dent	N/A	N/A	N/A	N/A	N/A	3.000
Int J Oral Max Surg	N/A	17 (13-23)	244 (175-280)	N/A	N/A	2.986
Int J Implant Dent	95 (84-107)	11 (11-25)	N/A	118 (109-129)	N/A	2.984
Oper Dent	N/A	390 (345-391)	N/A	N/A	N/A	2.937
Int J Comput Dent	N/A	N/A	N/A	N/A	N/A	2.923
Int J Oral Max Impl	N/A	N/A	N/A	N/A	N/A	2.912
Odontology	171 (109-172)	17 (7-20)	N/A	179 (129-222)	N/A	2.885
Med Oral Pathol Oral	56 (54-94)	179 (158-189)	N/A	252 (239-252)	N/A	2.883
Gerodontology	239 (183-251)	23 (18-49)	318 (312-336)	274 (201-288)	563 (501-575)	2.750
Brit Dent J	69 (57-87)	287 (275-291)	N/A	360 (351-374)	N/A	2.727
Int J Dent Hyg	124 (123-127)	16 (4-26)	76 (68-244)	130 (130-150)	192 (190-371)	2.725
Am J Orthod Dentofac	N/A	N/A	N/A	N/A	N/A	2.711
Angle Orthod	92 (92-123)	83 (72-99)	N/A	195 (175-195)	N/A	2.684
Braz Oral Res	133 (133-138)	21 (21-22)	158 (158-170)	154 (154-161)	296 (289-304)	2.674
Arch Oral Biol	123 (119-189)	5 (4-6)	9 (8-9)	137 (123-194)	138 (127-198)	2.640
Int Dent J	N/A	N/A	N/A	N/A	N/A	2.607
Orthod Craniofac Res	82 (61-92)	19 (14-54)	209 (188-230)	101 (101-106)	291 (291-329)	2.563
Or Surg Or Med Or Pa	115 (113-194)	6 (5-6)	253 (244-257)	121 (119-199)	370 (353-447)	2.538
Eur J Dent Educ	110 (95-152)	8 (7-9)	308 (308-311)	118 (101-162)	418 (403-475)	2.528
Commun Dent Oral	183 (140-184)	17 (16-30)	99 (99-208)	214 (152-227)	286 (249-336)	2.489
J Stomatol Oral Maxi	59 (56-91)	13 (9-16)	439 (427-440)	93 (65-104)	498 (496-515)	2.480
J Oral Facial Pain H	N/A	N/A	N/A	N/A	N/A	2.457
Dent Mater J	N/A	N/A	N/A	N/A	N/A	2.418
Pediatr Dent	132 (104-146)	N/A	N/A	N/A	N/A	2.378
J Orofac Orthop	215 (177-223)	74 (72-81)	N/A	251 (246-295)	N/A	2.341
Eur J Paediatr Dent	N/A	N/A	N/A	N/A	N/A	2.327
J Dent Educ	28 (24-40)	19 (13-22)	355 (337-358)	53 (35-53)	372 (371-389)	2.313
Aust Dent J	18 (18-18)	11 (8-12)	182 (180-191)	36 (36-36)	209 (209-209)	2.259
J Public Health Dent	196 (152-199)	15 (12-16)	305 (305-320)	208 (168-230)	501 (472-504)	2.258
Head Face Med	162 (139-390)	17 (15-26)	N/A	190 (165-407)	N/A	2.246
Acta Odontol Scand	181 (157-303)	25 (19-33)	N/A	214 (181-319)	N/A	2.232
Int J Peridont Rest	N/A	N/A	N/A	N/A	N/A	2.227
Qunitessence Int	N/A	N/A	N/A	N/A	N/A	2.175
Eur J Oral Sci	127 (88-134)	50 (43-131)	198 (170-199)	171 (131-272)	297 (293-333)	2.160
J Oral Maxil Surg	6 (2-48)	21 (15-23)	187 (174-189)	25 (23-73)	191 (189-207)	2.136
J Peridontal Implan	141 (134-146)	62 (62-68)	N/A	214 (196-232)	N/A	2.086
Brit J Oral Max Surg	N/A	270 (54-281)	362 (362-387)	N/A	N/A	2.018
J Adv Prosthodont	95 (60-136)	17 (16-28)	N/A	124 (77-152)	N/A	1.989
Cleft Palate-Cran J	N/A	N/A	N/A	N/A	N/A	1.915
Oral Radiol	113 (56-125)	41 (23-47)	N/A	139 (108-160)	N/A	1.882
IInt J Prosthodont	N/A	N/A	N/A	N/A	N/A	1.785
Am J Dent	N/A	N/A	N/A	N/A	N/A	1.748
Aust Endod J	255 (155-320)	45 (30-73)	394 (317-415)	327 (216-362)	606 (472-735)	1.719
Cranio	N/A	N/A	N/A	N/A	N/A	1.670
J Oral Sci	121 (106-151)	84 (79-88)	N/A	199 (168-230)	N/A	1.630
Oral Hlth Prev Dent	N/A	N/A	N/A	N/A	N/A	1.595
J Oral Implantol	N/A	N/A	N/A	N/A	N/A	1.546
Kor J Orthod	121 (69-129)	4 (2-6)	153 (150-169)	131 (69-140)	279 (212-289)	1.361
J Can Dent Assoc	/A	N/A	N/A	N/A	N/A	1.348
Semin Orthod	N/A	N/A	N/A	N/A	N/A	1.340
J Clin Pediatr Dent	N/A	N/A	N/A	N/A	N/A	1.338
Commun Dent Hlth	N/A	N/A	N/A	N/A	N/A	1.330
Australas Orthod J	N/A	N/A	N/A	N/A	N/A	0.269
Implantologie	N/A	N/A	N/A	N/A	N/A	0.127

**Figure 2:**
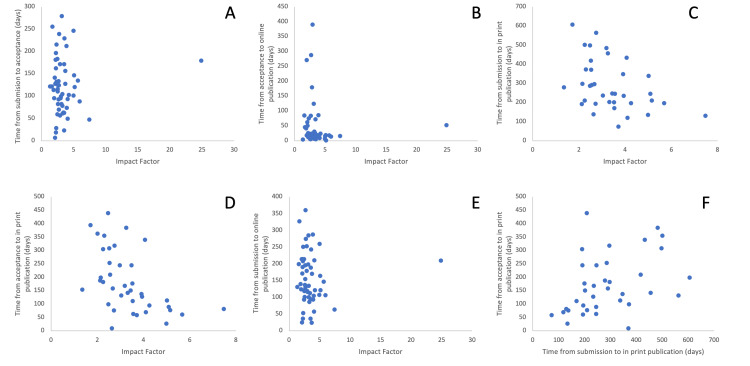
Scatterplots showing correlation between IF and publication times. Correlation between IF and time from  A) submission to acceptance (days) r = -0.063, p = 0.649; B) acceptance to online publication (days) r = -0.253, p = 0.055; C) submission to in print publication (days) r = -0.442, p = 0.007; D) acceptance to in print publication (days) r = -0.616, p <.001; E) submission to online publication (days) r = -0.156, p = 0.255; F) submission to in print publication and time from acceptance to in print publication (days) r = 0.475, p = 0.003 IF, impact factor

There were no correlations between IF and time from: submission to acceptance, acceptance to online publication, or submission to online publication (r = -0.063, p = 0.649; r = -0.253, p = 0.055; r = -0.156, p = 0.255, respectively). There were statistically significant correlations between time from: acceptance to in print publication, submission to in print publication, and IF (r = -0,616, p < 0.001; r = -0.442, p = 0.007, respectively). Journals with longer times from submission and acceptance to in print publication were associated with lower IF values. Figure 2 shows the relationship between IF and publication times.

## Discussion

Bibliometric analysis of scientific papers has a big importance for the research community. It is used by both researchers and institutions to estimate the time required for paper publication. This study was designed to evaluate publication times in peer-reviewed dentistry journals. Journals that published bibliometric data pretended to have higher IF values. The IF was negatively correlated with times from submission (r = -0.442, p = 0.007), and acceptance (r = -0.616, p < 0.001) to in-print publication.

The time of publication process is one of the most crucial indicators of a journal’s quality [1-2, 12]. The publication process relies on two intervals: time between the submission of a manuscript to acceptance which is spent on revisions and the peer review process, and the time from acceptance to publication in print which is usually consumed on editing, proofreading, typesetting, and printing [1-2]. From the authors' experience, time spent by researchers revising the manuscript is variable, depends on the current schedule and other duties thus may contribute to the overall delay in publication.

The present study is the first that focuses on publication time in dentistry journals. Both review-only journals and review articles were excluded because most of them are invited by editors and do not have to follow the strict timeline [1-2]. To maintain harmony in the selection process, only original articles were included. It was revealed that 34% of analyzed journals did not publish the dates of manuscript submission and approximately 50% of journals had no available publication times, which is much more when compared to other disciplines (e.g. 20% for ophthalmology, 15% for plastic surgery journals) [1-2]. In contrast to previous studies, journals that had available publication times tended to have higher IF values [1-2]. Although some journals do not provide information about the publication times of the manuscripts, approximate time for editorial decisions is posted on their websites, offering limited information about the publishing timeline.

In the present study, individual median peer-review time for dentistry journals varied widely, with an overall peer-review time of 115 days. In top plastic surgery journals in 2020, the median time from submission to acceptance was 5.4 months (approximately 162 days), and in ophthalmology journals was 119 days, which is comparable to dentistry journals [1, 13]. Significant time delay in the publication process could influence the reporting of significant clinical findings [2]. It was strongly documented that trials with positive findings were more likely to be published than those with negative or inconsistent results, therefore introducing a publication bias [2, 5, 14-16]. However, other authors did not find any significant associations between clinical trial outcomes and publication times [2, 6].

Higher IF values were associated with shorter times from submission (r = -0.442, p = 0.007), and acceptance (r = -0.616, p <.001) to in-print publication. This is in accordance with policies employed by highly cited journals [17-20]. It was revealed that journals speed up turn-around times and employ fast-track publication for potentially high-impact papers [17-20]. Medical scientists appear more reliant on print than electronic journals [21-24]. Some editors even deliberately personally approach lead investigators of the major research projects to get papers with the highest impact potential [17-20]. All of these are aimed to enhance the journal’s IF and do editorial duty.

There are some limitations in the present study. Authors did not have access to some journals which decreased the number of analyzed both journals and articles. A lower number of articles was analyzed than in the previous bibliometric analysis. Only five random original articles were extracted from each journal and no validated randomization method was utilized. Bibliometric factors such as the cited half-life of the journals and immediacy index are both less desirable by the authors and recognizable by the society [25-28], thus were excluded from the study. 

## Conclusions

To the knowledge of the authors, the present study is the first bibliometric analysis of the dentistry journals in the literature. It has the potential to be a valuable source of information for both authors and editors. Authors could easier select journals with short turn-around times and editors compare their journals with each other. Publication times availability was revealed to be an indicator of higher impacted journals, which is a potential new exponent of journal quality. Higher IF values were associated with shorter times from submission to acceptance and in-print publication, which is consistent with mentioned editorial policies.
